# Image intensifier distortion correction for fluoroscopic RSA: the need for independent accuracy assessment

**DOI:** 10.1120/jacmp.v13i1.3441

**Published:** 2012-01-05

**Authors:** Angela E. Kedgley, Anne‐Marie V. Fox, Thomas R. Jenkyn

**Affiliations:** ^1^ Department of Bioengineering Imperial College London, South Kensington Campus London SW7 2AZ United Kingdom; ^2^ Wolf Orthopaedic Biomechanics Lab, Fowler Kennedy Sport Medicine Clinic University of Western Ontario London Ontario N6A 5B9 Canada; ^3^ Department of Mechanical and Materials Engineering, Faculty of Engineering University of Western Ontario London Ontario N6A 5B9 Canada; ^4^ Graduate Program in Biomedical Engineering, Faculty of Engineering University of Western Ontario London Ontario N6A 5B9 Canada; ^5^ School of Kinesiology, Faculty of Health Sciences University of Western Ontario London Ontario N6A 5B9 Canada

**Keywords:** fluoroscopy, artifacts and distortion, reconstruction

## Abstract

Fluoroscopic images suffer from multiple modes of image distortion. Therefore, the purpose of this study was to compare the effects of correction using a range of two‐dimensional polynomials and a global approach. The primary measure of interest was the average error in the distances between four beads of an accuracy phantom, as measured using RSA. Secondary measures of interest were the root mean squared errors of the fit of the chosen polynomial to the grid of beads used for correction, and the errors in the corrected distances between the points of the grid in a second position. Based upon the two‐dimensional measures, a polynomial of order three in the axis of correction and two in the perpendicular axis was preferred. However, based upon the RSA reconstruction, a polynomial of order three in the axis of correction and one in the perpendicular axis was preferred. The use of a calibration frame for these three‐dimensional applications most likely tempers the effects of distortion. This study suggests that distortion correction should be validated for each of its applications with an independent “gold standard” phantom.

PACS numbers: 87.59.C, 87.57.cp, 87.57.nf

## I. INTRODUCTION

Fluoroscopic images, which are obtained using electron lenses, may suffer from all of the primary aberrations that affect images obtained through classical light optics,^(^ ^1^ ^)^ and correction of these aberrations has been the subject of many previous investigations.^(^ ^2^ ^–^ ^13^ ^)^ Distortion of the image is present in three distinct modes. “Pincushion” distortion is primarily caused by the process by which the electrons are focused onto a curved surface within the image intensifier, from which an image is then transferred to a flat plane image intensifier (II).^(^ ^3^ ^,^ ^14^ ^)^ It may be corrected to some extent within the II; although, due to the proprietary nature of such technology, it is difficult to know by how much it is corrected. S‐shaped, spiral, or pocket handkerchief distortion occurs primarily as a result of Earth's or other electronic devices' homogeneous magnetic fields,^(^ ^15^ ^)^ which are variable, and such distortion is dependent upon the orientation of the II. However, correction may be accomplished using shielding or a coil that creates a magnetic field in opposition to that created by Earth or other objects.^(^ ^3^ ^)^ Again, it is difficult to know the extent to which this has been corrected by the manufacturer. Localized distortions may also occur due to strong, inhomogeneous magnetic fields.

Distortion correction is most often performed with the help of a grid of wires or beads that is temporarily placed in front of the II to quantify the amount of distortion present. Either a local or a global approach to postprocessing distortion correction may be taken or, as reported in one case, local and global approaches have been combined.^(^ ^10^ ^)^ Local distortion correction algorithms use the coordinates of three or four grid points surrounding a small area of an image to correct for distortion within that area. This method is more susceptible to the influence of image noise or errors in digitization.^(^ ^5^ ^,^ ^7^ ^,^ ^10^ ^)^ In addition, a local correction scheme will produce discontinuities between each “cell” in the image.^(^ ^6^ ^,^ ^8^ ^)^ Global distortion correction algorithms use the coordinates of as many grid points as are visible in an image and calculate the distortion vector at each point. These data are then used to determine an overall expression for the distortion across the entire image. This may be calculated according to Cartesian^(^ ^2^ ^,^ ^5^ ^,^ ^6^ ^,^ ^7^ ^,^ ^10^ ^,^ ^12^ ^)^ or radial^(^ ^3^ ^)^ coordinates of the image. The positions of the beads in the image are generally related to the known positions of the beads according to a polynomial; however, in one reported case, thin‐plate splines were used.^(^ ^10^ ^)^ Cartesian polynomial fits are preferred, as distortion is usually nonradial, due to the effects of s‐shaped distortion.^(^ ^6^ ^)^ In a direct comparison of global and local techniques, the global technique was found to be superior.^(^ ^6^ ^)^

Most distortion correction in the literature is performed with diagnostic imaging applications in mind; that is, quantitative coronary angiography, three‐dimensional (3D) reconstructions, stereotaxic angiography, and radiotherapy treatment.^(^ ^7^ ^)^ To determine the optimal polynomial formulation to correct for distortion, a range of polynomial functions has been tested. The residuals and root mean squared (RMS) errors of the fit of the polynomial have been examined,^(^ ^3^ ^,^ ^8^ ^,^ ^11^ ^)^ and corrections have been tested on the same grid in a different position, or on a second grid.^(^ ^4^ ^,^ ^5^ ^,^ ^7^ ^,^ ^6^ ^,^ ^9^ ^,^ ^10^ ^,^ ^12^ ^)^ One study used a phantom in addition to other evaluation methods to quantify correction for digital tomosynthesis.^(^ ^11^ ^)^ However, no studies have determined the efficacy of distortion correction for radiostereometric analysis (RSA) using an independent phantom.

RSA is a technique by which the 3D positions of objects in space are reconstructed from information obtained by X‐ray imaging. A biplane fluoroscopy‐based RSA system^(^ ^16^ ^)^ was used for this study. Several studies have examined the overall accuracy of RSA systems, and it appears that accuracies are steadily improving with time.^(^ ^17^ ^–^ ^21^ ^)^ High accuracies mean smaller numbers of subjects are required in clinical studies, since significance may be reached more quickly.

The aim of this study was to compare the effects of a range of polynomials for distortion correction using a global approach by examining the fit of the chosen polynomial to the points on the distortion grid, the distances between the points of the grid in a second position, and the overall accuracy of the RSA reconstruction. It was hypothesized that the three outcome measures would lead to the same, most suitable, polynomial.

## II. MATERIALS AND METHODS

Two X‐ray fluoroscopes with 9‐inch image intensifiers (SIREMOBIL Compact‐L mobile C‐arms, Siemens Medical Solutions Canada Inc., Mississauga, Canada) were used in this study. These fluoroscopes are commonly used in concert to perform fluoroscopic RSA. The average pixel size of the IIs was quantified using a purpose‐made grid of 0.2 mm holes placed at the center of the IIs, and was found to be 0.3833 mm. In order to perform the distortion correction, a grid of 131 2 mm diameter stainless steel beads, with a spacing of 15mm×15mm, was created on a 9.5 mm thick Delrin sheet. The positions of these beads on the grid were precisely determined with the use of a coordinate measuring machine (CMM, DEA Swift, Hexagon Metrology Services Ltd., London, UK). The grid was rigidly attached to a ring that fit into a ridge in the housing of the IIs. Following each RSA testing session, the grid was placed over each II and an image was obtained of the distorted positions of the grid beads.

The positions of the beads in each fluoroscopic image were manually located using a mouse and custom‐written software (MATLAB, The MathWorks Inc., Natick, USA), which applied a pixel‐weighting algorithm similar to those described in the literature.^(^ ^6^ ^,^ ^9^ ^,^ ^12^ ^)^ Distortion correction was performed using a global approach with a range of polynomial fits, from first degree in each direction (a second order polynomial) through to third degree in each direction (a sixth order polynomial). Polynomial coefficients were determined based on a least squares minimization. Two polynomials were defined for each correction, one for correction in the x‐axis of the image (horizontal within the image) and one for correction in the y‐axis of the image (vertical within the image). Polynomials were paired for correction such that the order in x for the x‐axis was the same as the order in y for the y‐axis and vice versa, as this yielded the best results. Therefore, for example, if a polynomial of third order in x and first order in y was used to correct the x‐coordinate, then a polynomial of first order in x and third order in y was used to correct the y‐coordinate. The number of coefficients in the polynomial dictated the minimum number of beads that were required for a solution. For instance, a polynomial with a maximum degree of one in each of x and y has four coefficients. It has been demonstrated that for good distortion correction results, the number of beads in the distortion grid needs to be at least a factor of three more than the number of coefficients in the correction scheme.^(^ ^5^ ^)^ Therefore, in this simple case, the grid would need to have at least twelve beads. With 131 beads, the grid was more than sufficient to correct for our highest order polynomial, which had 16 coefficients.

To evaluate the correction of the polynomials in isolation, the distortion grid was oriented on each II as it would be done following an RSA session. The polynomial coefficients and the RMS errors of the fits were determined for each polynomial. The grid was then replaced on the II in a second location, such that the beads were translated to an intermediate, staggered location between the original positions. Distances between the beads were calculated and compared to those measured with the CMM, following correction with the various polynomials. This was done with each of the two IIs in six different positions and orientations, covering the full range of their motion.

A previously described fluoroscopy‐based RSA system was used.^(^ ^16^ ^)^ Images from the fluoroscopy units were 720 pixels by 540 pixels. Image processing and RSA reconstruction were performed with software custom‐written in MATLAB.^(^ ^16^ ^)^ A custom‐designed calibration frame was employed.^(^ ^22^ ^)^ The two fluoroscopes were positioned approximately orthogonal to one another. An accuracy phantom consisting of four 1 mm stainless steel beads mounted on carbon fiber rods fixed securely to a wooden base^(^ ^16^ ^)^ was placed in the capture volume. The distances between each pair of beads ranged from 25.11 mm to 41.95 mm, as determined using a coordinate measuring machine (CMM, Model MDX‐20, Roland DG Corporation, Hamamatsushi, Japan; scanning pitch: 0.05 mm in x and y and 0.025 mm in z). Forty pairs of images were obtained without moving the phantom. This was followed by 16 pairs of images where the phantom was reoriented in position and rotated within the field of view in a randomly selected manner between each set. These poses ranged across the full area of both of the IIs. The X‐ray tube voltage and current were set automatically by the fluoroscopy units and were 51 kVp and 0.4 mA for the calibration frame and 49 kVp and 0.3 mA for the phantoms.

The primary measures of interest were the distances between the four beads of the phantom as measured using RSA, with image distortion correction provided by the range of polynomial fits. These distances were measured and compared with the distances obtained using the CMM. An overall error for the RSA reconstruction of each position was calculated by averaging the errors in all the distances. Accuracy was defined as the average error in the reconstruction of the phantom over the 16 trials with motion between them. Precision was defined as the standard deviation of the error in the reconstruction of the phantom over the 40 static trials. Secondary measures of interest were the RMS error of the fit of the chosen polynomial to the distortion grid points and the errors in the distortion corrected distances between the points when the grid was replaced on the II.

Statistical analysis of the RMS errors of the fits of the various polynomials, the distances between the beads, and the accuracy of the RSA reconstructions were performed using Friedman Repeated Measures Analyses of Variance on Ranks followed by post hoc Student‐Newman‐Keuls multiple comparisons. These were also used to compare the results between the two fluoroscopes. Levene's test was used to analyze the precision data to determine any differences in the standard deviations of the RSA reconstructions. Statistically significant differences were defined as p<0.05.

## III. RESULTS

**Figure 1:**
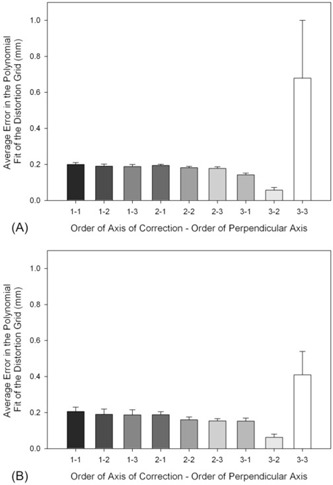
RMS errors of the various polynomial models that were employed in (A) the x‐direction and (B) the y‐direction (mean ±1 standard deviation). All corrections were found to be different from 3−2(p<0.05). Lighter bars indicate higher order polynomial models.

No significant differences were found between the two fluoroscopes in the RMS errors as a function of II orientation and polynomial degree. Therefore, the results for the two fluoroscopes were combined to compare the effects of the various polynomial corrections. RMS errors ranged from 58±15μm in the x‐direction and 63±18μm in the y‐direction, for a fifth order polynomial of third order in the axis of correction and second order in the perpendicular axis (i.e., for correction of the x‐coordinates of points in the image, the polynomial would be of third order in x and of second order in y), to 679±321μm in the x‐direction and 410±130μm in the y‐direction, for a third order polynomial in each direction (Fig. 1). Significant differences were found between distortion correction using a polynomial of order three in the axis of correction and two in the perpendicular axis and all other orders of correction tested (p<0.05).

**Figure 2:**
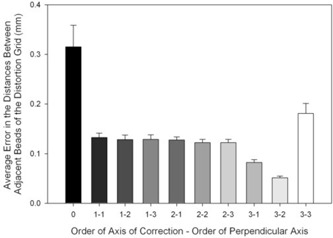
Errors in the distances between adjacent beads (mean (±1 SD) of the distances measured on a second staggered image of the same grid and plotted as a function of the polynomial correction). All corrections were found to be different from 3−2(p<0.05). Lighter bars indicate higher order polynomial models.

Similar results were obtained when the distances between the beads of the grid in a second, staggered position were examined (Fig. 2). Errors in uncorrected points were 315±43μm. Average errors in corrected pointed ranged from 51±3μm, for a fifth order polynomial of third order in the axis of correction and second order in the perpendicular axis, to 181±21μm, for a third order polynomial in each direction. The use of a polynomial of order three in the axis of correction and two in the perpendicular axis was found to result in significantly lower errors than all other orders of correction tested (p<0.05).

**Figure 3:**
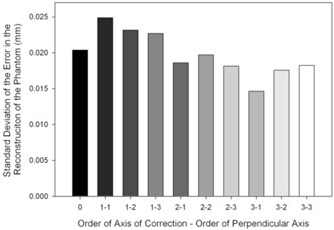
Precision of the reconstruction (defined as the standard deviation of the error in the reconstruction of the phantom over the 40 static trials). Lighter bars indicate higher order polynomial models.

The precisions of the reconstructions with each set of polynomials are shown in Fig. 3. They ranged from 14.6 μm to 24.9 μm, with the former using a polynomial of order three in the axis of correction and one in the perpendicular axis and the latter using a first order polynomial in each direction. There were no differences in the precisions of the reconstructions between any of the polynomial corrections.

**Figure 4:**
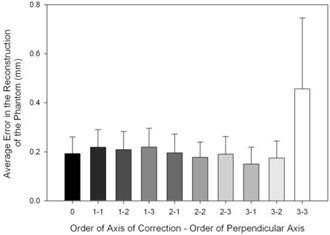
Accuracy of the reconstruction (mean (±1 SD) of the error in the reconstruction of the phantom over the 16 trials with motion between them). All corrections found to be different from 3−1(p<0.05). Lighter bars indicate higher order polynomial models.

Average accuracies of the RSA reconstructions ranged from 150±69 μm, for a polynomial of third order in the axis of correction and first order in the perpendicular axis, to 457±289 μm for a third order polynomial in each direction (Fig. 4). RSA reconstruction of the uncorrected points resulted in an error of 193±68 μm. The use of a polynomial of order three in the axis of correction and one in the perpendicular axis was found to have significantly lower errors than all other orders of correction tested (p<0.05). First order polynomials in the axis of correction always resulted in worse errors than the uncorrected points (p<0.05), regardless of the order of correction in the perpendicular axis. Third order polynomials in each axis also resulted in worse errors (p<0.05).

## IV. DISCUSSION

Contrary to our hypothesis, the polynomial with the smallest RMS error in solving for the polynomial coefficients and the smallest errors in the distances between points on the translated distortion grid did not have the lowest average RSA reconstruction error. This is similar to the outcome of the only other study to employ a 3D phantom to quantify the results of distortion correction, where it was found that a fifth order correction resulted in the best polynomial fit.^(^ ^11^ ^)^ However, as the images were being used for digital tomosynthesis, the variability of the correction coefficients with the fifth order was a concern. Therefore, a fourth order was preferred. In this investigation, the variability of the RMS error of the fit of the sixth order polynomial to the distortion grid points would be a concern, as indicated by the high standard deviations in Fig. 1 (bar 3–3).

It had been found that distortion correction requirements may vary between IIs, although this was tested for four different models of two different makes, and not various IIs of the same make and model.^(^ ^3^ ^,^ ^6^ ^)^ Using the two IIs of our system it was found that, as would be expected, distortion correction requirements did not differ.

In this study, the most appropriate choice of polynomial for distortion correction was not found to be dependent upon II orientation. Nevertheless, it was found to vary depending upon the intended use of the images. Although most applications of IIs are two‐dimensional, it is increasingly common to use IIs for 3D analyses. The use of a calibration frame for these 3D applications most likely tempers the effects of distortion — leading to accuracies in the RSA reconstruction using uncorrected points that were far better than anticipated (as in Fig. 4). The distances between the beads in two dimensions indicate that distortion correction plays a much larger role in two‐dimensional measurements. In addition, as anticipated, the precision of the measurements was not affected by distortion correction.

The advantages and disadvantages of the various methods of distortion correction have been examined in the literature, and it is generally agreed that the main disadvantage of implementing a global approach is that any extreme local distortions cannot be corrected;^(^ ^5^ ^)^ however, none are to be expected. The advantages are the decreased susceptibility to image noise and the ability to extrapolate the correction beyond the boundaries of the calibration points.^(^ ^5^ ^)^ It has been shown that higher order polynomial corrections tend to result in higher RMS errors, as any noise that is present is modeled in addition to the distortion.^(^ ^6^ ^,^ ^10^ ^)^ This may explain why errors from the third order polynomial were so high. Nevertheless, the chosen polynomial must have a sufficient degree to model the distortion; therefore, employing a first order polynomial in the axis of correction was insufficient, as demonstrated in Fig. 4.

This study had the same potential sources of error as those that are present when working with any fluoroscopy‐based RSA system. These potential sources include image distortion and bead blurring due to the stochastic nature of X‐ray detection. Image distortion, being the subject of this study, has clearly been addressed above. Bead blurring is addressed through the pixel‐weighting bead location algorithm. In addition, the RSA algorithm has several built‐in methods of decreasing the effects of other system noise, including a least squares method of reconstruction.^(^ ^16^ ^)^

## V. CONCLUSIONS

The findings of this study suggest that while significant efforts have been made to generalize distortion correction for IIs, correction should be validated with an independent “gold standard” phantom for each particular application for which it is to be used, as one correction algorithm may not suffice for all applications.

## ACKNOWLEDGMENTS

The authors would like to thank the Natural Science and Engineering Research Council (NSERC) and the Canadian Foundation for Innovation for their financial support.
